# TRIM8 anti-proliferative action against chemo-resistant renal cell carcinoma

**DOI:** 10.18632/oncotarget.2081

**Published:** 2014-06-08

**Authors:** Mariano Francesco Caratozzolo, Alessio Valletti, Margherita Gigante, Italia Aiello, Francesca Mastropasqua, Flaviana Marzano, Pasquale Ditonno, Giuseppe Carrieri, Hélène Simonnet, Anna Maria D'Erchia, Elena Ranieri, Graziano Pesole, Elisabetta Sbisà, Apollonia Tullo

**Affiliations:** ^1^ Institute for Biomedical Technologies ITB, Bari, Italy; ^2^ Institute of Biomembranes and Bioenergetics IBBE, Bari, Italy; ^3^ Dept Biomedical Science, University of Foggia, Foggia, Italy; ^4^ Dept Biosciences, Biotechnologies and Biopharmaceutics, University of Bari “A. Moro”, Bari, Italy; ^5^ Dept Emergency and Organ Transplantation DETO, University of Bari “A. Moro”, Bari, Italy; ^6^ Dept Surgical Science, University of Foggia, Foggia, Italy; ^7^ Centre de Recherche en Cancérologie de Lyon, Faculté de Médecine Lyon-Est, LYON Cedex 08 France

**Keywords:** ccRCC, drug resistance, TRIM8, cisplatin, nutlin 3, p53

## Abstract

In some tumours, despite a wild-type p53 gene, the p53 pathway is inactivated by alterations in its regulators or by unknown mechanisms, leading to resistance to cytotoxic therapies. Understanding the mechanisms of functional inactivation of wild-type p53 in these tumours may help to define prospective targets for treating cancer by restoring p53 activity.

Recently, we identified TRIM8 as a new p53 modulator, which stabilizes p53 impairing its association with MDM2 and inducing the reduction of cell proliferation.

In this paper we demonstrated that TRIM8 deficit dramatically impairs p53-mediated cellular responses to chemotherapeutic drugs and that TRIM8 is down regulated in patients affected by clear cell Renal Cell Carcinoma (ccRCC), an aggressive drug-resistant cancer showing wild-type p53. These results suggest that down regulation of TRIM8 might be an alternative way to suppress p53 activity in RCC. Interestingly, we show that TRIM8 expression recovery in RCC cell lines renders these cells sensitive to chemotherapeutic treatments following p53 pathway re-activation.

These findings provide the first mechanistic link between TRIM8 and the drug resistance of ccRCC and suggest more generally that TRIM8 could be used as enhancer of the chemotherapy efficacy in cancers where p53 is wild-type and its pathway is defective.

## INTRODUCTION

Conventional therapeutic protocols treat aggressive cancers causing massive DNA damage in order to induce apoptosis of the rapidly multiplying cancer cells. This strategy works for many cancers, in particular those which express wild-type p53 tumour suppressor protein as the efficacy of most chemotherapeutic drugs is dependent on a successful execution of p53-mediated apoptosis to override self-sufficiency in growth signals and insensitivity to antigrowth signals typical of cancer cells [1-3]. Indeed, tumours harbouring p53 mutations, which lead to expression of inactive p53 protein, account for about 50% of all human cancers. These tumours are associated with chemo resistance and, in general, predict a considerably worse patient prognosis in comparison with malignancies with functional p53 [4-6]. Besides the many tumours that have an inactivating mutation in the TP53 coding sequence, an additional 40% do contain a wild-type TP53 gene but the p53 pathway is often inactivated through alterations in its regulators or rather still unknown mechanisms [7]. The reactivation of p53 in cancer cells is certainly a promising treatment strategy [8]. Current p53-based therapeutic attempts focus on ectopically expressing wild-type p53 in p53-null tumours or restoring p53 pathway in tumours in which p53 is incapacitated by alterations of other pathway components [9]. Among cancers showing rare p53 mutations and poor response to conventional anti-cancer treatments, renal cell carcinoma (RCC) represents an extraordinary example of the importance of p53 pathway alterations in therapy resistance. RCC is a family of cancers including five major subtypes (clear cell, papillary type I and type II, chromophobe, collecting duct, and unclassified RCC) that originate from the renal tubular epithelium, but unlike other epithelial cancers originating from other districts such as colon, breast, lung, stomach and bladder, p53 mutations in RCC are particularly rare, especially in the clear cell subtype (ccRCC) [10-13]. Resistance against most chemotherapeutic agents is partly mediated by multidrug resistance protein 1 (MDR1) whose expression is higher in the invasive tumours (e.g., ccRCCs) compared with non-invasive kidney tumours, such as renal oncocytomas (ROs) and decreases in the more undifferentiated tumours, but still remains at levels high enough to be drug resistant [14, 15]. Besides this, resistance against radiotherapy and cytotoxic drugs depends on the disruption of p53 signalling, although the importance of p53 alterations in RCC has been the subject of conflicting observations [16]. However, several studies showed that mutated p53 found in the papillary, chromophobe, and ccRCC subtypes, appears to be accompanied by metastatic progression of the disease and poor survival of patients with RCC and a correlation of p53 expression with TNM classification seems to suggest that p53 mutations might have an important role in the progression of RCC [17-19]. The induction of the p53 pathway during treatment of RCC clearly could boost the cellular response to stress stimuli led by cytotoxic chemotherapy regimens, normally ineffective on the most aggressive subtype of this family of cancers, i.e., ccRCC. Also, immunotherapy with interferon alpha (INFα) and interleukin 2 (IL2), the first therapeutics to result in regressions of metastatic renal cell carcinoma, seems to take advantage of a reactivation of p53. Indeed, these biological response modifiers, as these cytokines are often defined, predominantly act through a combination of the stimulation of cell-mediated cytotoxicity, a direct antiproliferative activity, and anti-angiogenetic effects, in which p53 may be involved considering its role in controlling cell growth, apoptosis and angiogenesis [20, 21].

In this complex scenario of p53 stability and activity regulation, we recently identified a new key regulator of p53 in the cell cycle arrest versus apoptosis decision, i.e. TRIM8 [22]. We demonstrated that p53 promotes the transcription of TRIM8, which in turn is able to interact with p53 preventing the binding to MDM2 hence resulting in the stabilization of p53. To further strengthen these recent findings, TRIM8 was found to induce also the degradation of MDM2. Interestingly, TRIM8 deficit dramatically impaired p53 stabilization and activation after a genotoxic stress.

TRIM8 belongs to a subfamily of the RING type E3 ubiquitin ligases characterized by a tripartite motif and some of whose members function as important regulators for carcinogenesis [23]. Each member seems to have a peculiar function relative to the p53 pathway, both antagonizing and enhancing p53 response to specific stimuli [24-26].

Here we show that TRIM8 deficit dramatically impairs p53 stabilization and activation in response to chemotherapeutic drugs. By comparing tumour and corresponding healthy tissues, we found that in patients affected by ccRCC, TRIM8 expression level is decreased, while no alterations were observed in ROs. Importantly, the restoration of TRIM8 levels in RCC cell line makes them more sensitive to the action of Nutlin-3 and Cisplatin treatments, through the reactivation of the p53 pathway.

## RESULTS

### TRIM8 silencing prevents p53 activation after chemotherapeutic drug treatment

**Figure 1:**
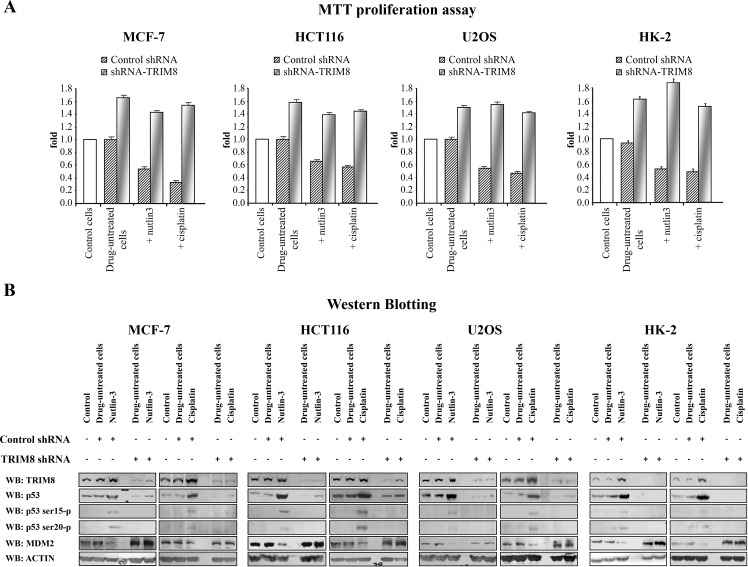
TRIM8 silencing prevents p53 activation after chemotherapeutic drug treatment Cell proliferation by MTT reduction assay (A) and protein levels of the indicated proteins by Western blotting analysis (B) were measured in the indicated cell lines in control cells, 48h after transfection with unspecific shRNA or specific TRIM8-shRNAs (drug-untreated cells) and 24h after chemotherapeutic drug treatment (Cisplatin 7.5 μM or Nutlin-3 10 μM). Western blot of Actin was conducted as control.

In order to test the effects of TRIM8 deficit on the p53-dependent cellular response to chemotherapeutic drugs, different p53 wild-type cell lines (HCT116, MCF-7, U2OS, HK-2) were transfected with unspecific shRNA control vector or with TRIM8 specific shRNA. The abrogation of TRIM8 endogenous expression was confirmed by western blotting and qRT-PCR (Figure 1B and Supplementary Figure 1). After transfection the cells were treated with two chemoterapeutic drugs, namely Nutlin-3 and Cisplatin. MTT proliferation assays demonstrated that TRIM8 silencing resulted in increased cell proliferation rate as a result of MDM2 protein stabilization and accordingly p53 protein degradation in all cell lines analysed, impairing the anti-proliferative action of the chemotherapeutic drugs (Figure 1A-B). As it is well known that Cisplatin and Nutlin-3 induce p53 activation [27-31], we analysed whether TRIM8 deficit could impair this activation. We found that in MCF-7, HCT116, U2OS and HK-2 cell lines, Nutlin-3 as well as Cisplatin treatment induced p53 stabilization and transactivation of p53 target genes (e.g. p21, BAX, GADD45) (Figure 1B and Supplementary Figure 2), while TRIM8 silencing brought down p53 endogenous protein levels and the transactivation of p53 target genes involved in cell cycle arrest program (p21 and GADD45) (Figure 1B and Supplementary Figure 2) [22]. Consistently, TRIM8 depletion prevented p53 phosphorylation on Ser-15 and Ser-20 upon drug treatments as demonstrated by the addition of proteasome inhibitor MG132, which preserved the degradation of p53 and clearly indicated that p53 is not phosphorylated (Figure 1B and Supplementary Figure 3). Consistent with these results, we found that TRIM8 silencing induced MDM2 stabilization, thereby impairing Cisplatin and Nutlin-3 effect (Figure 1B).

These results strongly suggest that TRIM8 levels are relevant to the p53-mediated cellular responses to chemotherapeutic drugs.

### TRIM8 expression is down regulated in ccRCC

In the attempt to translate our findings *in vivo* and set the basis for the use of TRIM8 as enhancer of the chemotherapy efficacy, we investigated whether in tumours highly resistant to chemotherapy, TRIM8 expression levels were lower compared to normal tissue. Thus we measured TRIM8 expression levels in patients affected by clear cell Renal Cell Carcinoma (ccRCC) or renal oncocytoma (RO). We considered these two subtypes of RCC as they show very different clinical behaviour, the first being extremely non responsive to conventional radiation and chemotherapeutic treatments, while the latter showing excellent prognosis because of its benign nature.

**Figure 2:**
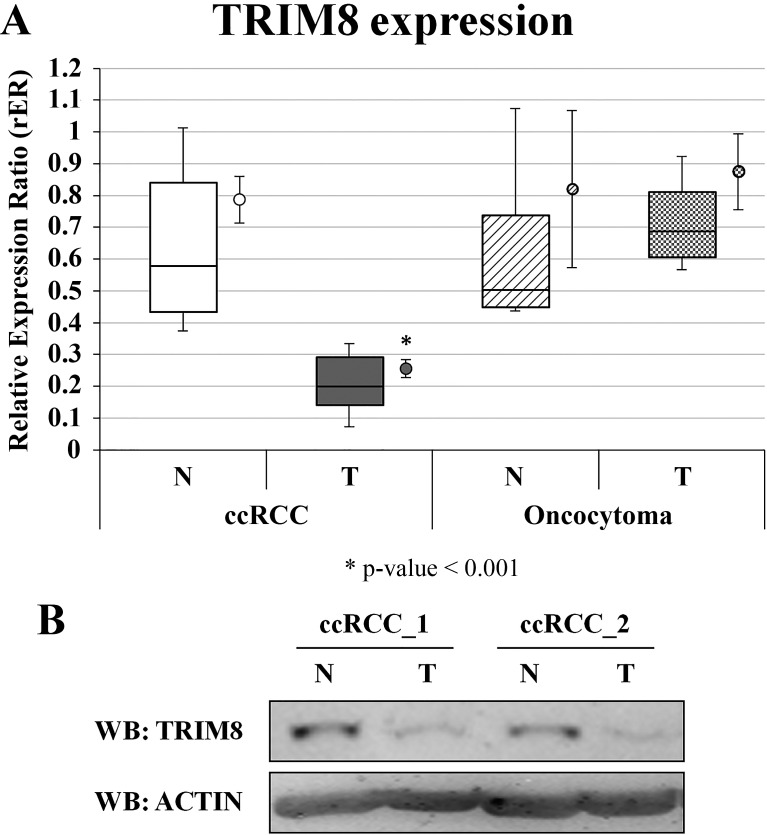
TRIM8 expression in renal cancer samples (A) TRIM8 mRNA expression in 20 ccRCC and 4 renal oncocytoma samples and their paired non-tumour tissues. Data are represented in box-plots showing median and 10^th^, 25^th^, 75^th^ and 90^th^ percentiles for each category of sample. The average expression (± standard error) is also reported as a dot on the right of the boxes. Expression data were measured respect to one normal sample chosen arbitrarily as calibrator and then normalized by the geometric mean of ACTB e RPL13 expression ratios. * p-value < 0.001. (B) Western blotting analysis of TRIM8 of two ccRCC patients representative of all.

Twenty patients (10 males and 10 females; mean age: 63.8 ± 10.8 years), who underwent surgery for ccRCC at histological analysis, and 4 patients (all males; mean age: 63.25 ± 4.86) affected by RO were analysed for TRIM8 expression by qRT-PCR and western blotting (Figures 2A-B). The relatively low number of RO samples was due to the rarity of occurrence of this benign neoplasia. Two samples from each patient were available, one from renal cancer tissue and one from non-neoplastic surrounding renal epithelial tissue.

As shown in Figure 2A, on average tumour samples expressed TRIM8 at a lower level (3.2-fold; p-value = 2.33E-06) than non-tumour renal epithelial tissue. Accordingly TRIM8 protein levels in tumour samples were lower compared to non-tumour counterpart (Figure 2B). Intriguingly, this is true for all the ccRCC sample pairs analysed. Considering the variation of TRIM8 expression in each ccRCC tissue pair and Fuhrman grade, no variation was detected indicating that the down-regulation of TRIM8 expression seems to be independent from the severity of this type of tumour (Supplementary Figure 4A). Importantly, no alterations in TRIM8 expression were observed in renal oncocytoma samples compared to non-tumour tissue (Figure 2A and Supplementary Figure 4B).

Since we previously demonstrated that TRIM8 is a direct p53 target gene [22], we investigated whether the decreased expression of TRIM8 in ccRCC was due to p53 mutations. Full-length p53 cDNA was amplified by PCR starting from total RNA extracted by tumour and non-tumour tissues. Sequence analysis showed that all the ccRCC and oncocytoma samples had wild-type p53 (data not shown). This finding was consistent with literature data indicating that the p53 gene is infrequently mutated in kidney cancers.

These results suggest that TRIM8 expression is down regulated in ccRCC but not in the benign oncocytoma, suggesting that the decrease of TRIM8 expression is linked to a malignant transformation of the cells.

### TRIM8 up-regulation restores p53 tumour suppressor activity in renal cell carcinoma

In order to rule out a potential role for TRIM8 deficit in determining the resistance of the ccRCC to chemotherapy due to prevention of p53 full activation, we evaluated whether the recovery of TRIM8 expression levels renders the renal tumour cells more sensitive to conventional chemotherapy.

**Figure 3:**
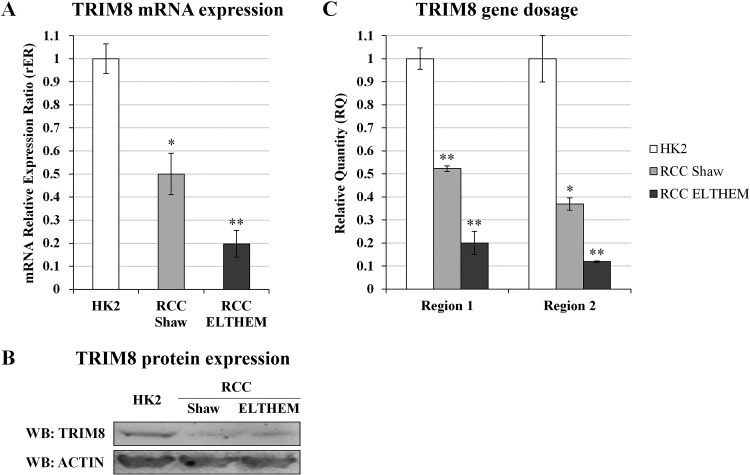
TRIM8 expression and gene dosage analysis in RCC cells (A) TRIM8 mRNA expression in HK-2 and RCC cell lines (Shaw and Elthem). The average expression (± standard deviation) is normalized to RPL13 expression level. (B) Western blotting analysis of TRIM8 of HK2 and RCC cell lines (Shaw and RCC ELTHEM). (C) TRIM8 gene dosage in HK-2 and RCC (Shaw and Elthem) cell lines. The ratio of qPCR signals from both TRIM8 region 1 and 2, and TP63 locus in HK-2 was normalized to 1.0 and used to calibrate the same ratios in RCC Shaw and Elthem. Data shown are the mean of at least three independent experiments. * p-value < 0.05. ** p-value < 0.01.

We took advantage of an immortalized proximal tubule epithelial cell line derived from normal adult human kidney (HK-2) and of two renal clear cell carcinoma derived cell lines (RCC Shaw and Elthem). HK-2 cells, already used for TRIM8 silencing experiments (Figures 1A-B and Supplementary Figure 1), retain functional characteristics of proximal tubular epithelium. All cell lines express wild-type p53. We first measured the TRIM8 expression levels in the three cell lines (Figure 3A-B) and confirmed by qRT-PCR and western blotting that RCC derived cell lines had lower TRIM8 mRNA and protein levels than HK-2, consistently with the results observed in RCC patients analysed (Figure 2A-B). Exploring the molecular basis of TRIM8 expression deficit observed in a p53 wild-type RCC cell lines, we analysed by quantitative PCR in these cell lines two regions of the TRIM8 gene and a region of the bi-allelic p63 gene locus as control. As expected, the p63 gene locus showed nearly identical quantification cycles (data not shown). After normalizing gene dosage data and comparing them to HK-2 cells, we observed a reduction of the 5′ region of TRIM8 gene (region 1) equal to about 0.5 in RCC Shaw cells and 0.2 in RCC Elthem cells, and similar ratios were calculated for the 3′ region (region 2), i.e., about 0.4 and 0.1, respectively in RCC Shaw and Elthem cell lines (Figure 3C). This clearly suggests that TRIM8 expression deficit in RCC cell lines could be due to the loss of one copy of the gene, although we cannot exclude other possible mechanisms.

**Figure 4:**
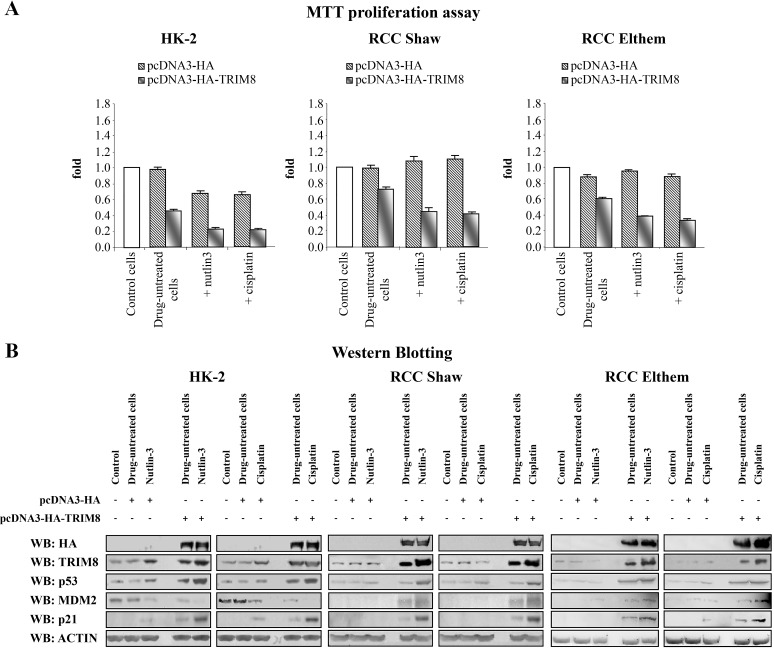
TRIM8 up-regulation restores p53 tumour suppressor response to chemotherapeutic drug treatments in renal cell carcinoma Cell proliferation by MTT reduction assay (A) and protein levels of the indicated proteins by Western blotting analysis (B) were measured in the renal cell lines HK-2, RCC Shaw and RCC Elthem (control), 48h after transfection with pcDNA3-HA control vector or pcDNA3-HA-TRIM8 (drug-untreated cells) and 24h after chemotherapeutic drug treatment with Cisplatin (7.5 μM) or Nutlin-3 (10 μM). Western blot of Actin was conducted as control.

Next, we analysed the response of these three cell lines to Cisplatin and Nutlin-3 treatment (Figures 4A-B). MTT proliferation assays demonstrated that Cisplatin and Nutlin-3 induced a reduction of HK-2 cell proliferation rate, but had no effect at all on RCC cell proliferation rate. Interestingly, the overexpression of TRIM8 in all cell types induced a great reduction in proliferation rate, which became more pronounced when the cells were treated with Nutlin-3 and Cisplatin (Figure 4A).

**Figure 5:**
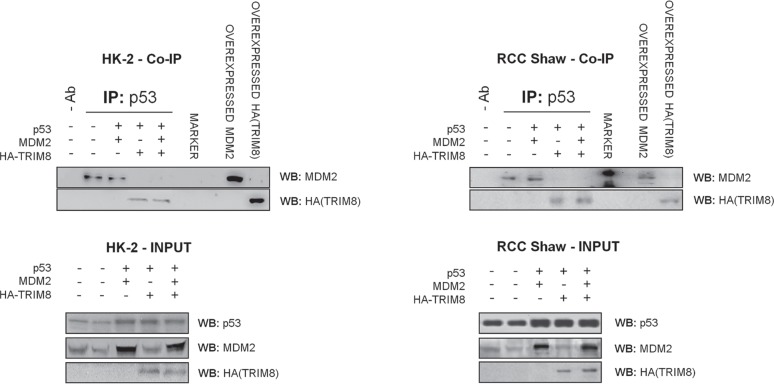
TRIM8 displaces MDM2-p53 binding HK-2 and RCC Shaw cells were co-transfected with the indicated plasmids. Cell lysates were subjected to immunoprecipitation with the p53 specific antibody DO-1. The immunoprecipitated complexes were analyzed by immunoblotting using MDM2- and HA- antibodies to detect respectively MDM2 and TRIM8 physical interaction with p53.

Next we investigated if the cell proliferation decrease observed in HK-2 and RCC lines, upon TRIM8 overexpression, was the result of p53 activation. Consistent with MTT results, only HK-2 cell line showed a faint p53 and p21 protein levels increase upon Nutlin-3 or Cisplatin treatment, while TRIM8 overexpression induced in all cell lines the stabilization of endogenous p53 and p21 proteins, whose levels were further increased after Cisplatin and Nutlin-3 treatment (Figure 4B). Interestingly, differently from HK-2 cells where in parallel with p53 stabilization, MDM2 protein levels decreased, in both RCC cell lines TRIM8 over-expression induced a stabilization of MDM2 (Figure 4B), though this stabilization did not lead to p53 degradation. Currently, we do not know how MDM2 increases in RCC cell lines upon TRIM8 overexpression. In order to understand the mechanism by which MDM2-mediated p53 degradation is prevented in RCC cells upon TRIM8 over-expression, we performed co-immunoprecipitation experiments, which demonstrated that MDM2-p53 binding is easily displaced when TRIM8 is expressed (Figure 5), as we previously found in HCT116 cells [22].

Altogether, these findings demonstrated that Cisplatin and Nutlin-3 treatments resulted more effective when TRIM8 expression is restored in ccRCC cells.

## DISCUSSION

ccRCC is the most common subtype of RCC accounting for about 80% of surgical cases and is characterized by exceptionally high resistance to radiation and chemotherapy despite harbouring a wild-type p53. Conflicting results have been reported on the functionality of p53 in RCC. One observed mechanism to hamper p53 function is the amplification of its negative regulator MDM2, detected in about 7% of all human cancers [32]. Although amplification of MDM2 is observed both in wild type and mutant p53 tumours, a significant correlation is found in tumours with p53 wild-type status [32]. In addition to MDM2 amplification, deletion of ARF, also resulting in elevated levels of MDM2, was found to reduce p53 function [33, 34]. In this context some authors postulated that p53, or more precisely its transcriptional activity, is intact in RCC, and that MDM2 alone should play a pivotal role in treatment resistance [35]. On the contrary, it has been reported that p53 activity is abolished in RCC cell lines independently from MDM2 by an uncommon dominant mechanism [36, 37].

In this rather complex scenario, we assessed whether TRIM8 deficit would contribute to the p53 inactivity following chemotherapeutic treatment. TRIM8 belongs to the Tripartite Motif protein family, whose members have been implicated in a variety of processes like development, differentiation and cancer [38, 39]. Recently, we demonstrated that under a stress condition p53 promotes the transcription of TRIM8, which in turn by a positive feedback mechanism interacts with p53, sustains its stabilization displacing MDM2, and promotes p53-dependent cell growth arrest [22]. Here we showed that TRIM8 deficit prevented p53 activation following drug treatments in several p53 wild-type cell lines (Figure 1B and Supplementary Figures 1, 2). Accordingly, we found that TRIM8 expression was dramatically down regulated in ccRCC (Figure 2A-B). This deficit is likely due to the loss of one copy of the gene (Figure 3C). It has been previously demonstrated that TRIM8 maps on chromosome 10q24.3 within a region mostly involved in deletions and rearrangements in brain tumours [40]. We cannot definitely assert that TRIM8 deficit in Renal Cell Carcinoma is specifically due to the loss of TRIM8 heterozygosity, as other additional mechanisms, such as epigenetic silencing, could be involved. In any case, since very low percentages of RCC cases are found to have p53 mutations, decreased expression of TRIM8 could be another mechanism to inhibit p53 function in RCC. Interestingly, TRIM8 expression recovery makes the RCC cells more sensitive to chemotherapy (Figure 4A-B). The molecular mechanism of such recovery relies on a TRIM8-mediated displacement of MDM2 from p53 (Figure 5), thus preventing p53 proteasome degradation. In RCC cell lines, differently from other cell lines [22], TRIM8 did not promote MDM2 degradation, involving other unknown mechanisms (Figure 4B). In this case it is plausible to envision that when TRIM8 is expressed in cells and supports p53 activation and phosphorylation upon genotoxic stimuli, MDM2 may not preside to its degradation any longer and be totally re-directed towards other oncogenic targets. Indeed, there is a variety of molecular targets that have been identified because of their altered expression levels in ccRCC, which may be important for tumour development and/or progression, thus becoming potential targets for specific therapies created to combat these alterations. Not surprisingly, some known molecular markers of ccRCC include hypoxia-inducible factors (HIFs) - that in normal conditions are negatively regulated by VHL (von Hippel-Lindau) tumour suppressor protein and positively by the mammalian Target of Rapamycin (mTOR) [41, 42] - and their transcriptional targets, notably those encoding vascular endothelial growth factor (VEGFA), transforming growth factor α (TGFα), platelet-derived growth factor (PDGF), epidermal growth factor receptor (EGFR), all involved in cell proliferation and survival [43, 44]. The hypothesis that MDM2 activity, after TRIM8 over-expression, could be directed towards oncogenic targets is supported by the finding that p53 promotes MDM2-mediated HIF1α degradation [45-48]. Some of the products encoded by these genes represent the main target of new therapies aimed at inhibiting tumour growth acting mainly on angiogenesis and mTOR pathway.

Many researchers have suggested the strategy of reactivating p53 in cancer cells as a promising treatment option [8]. Current p53-based therapeutic strategies focus on ectopic expression of wild-type p53 in p53-null tumours or restoration of the p53 pathway in tumours in which p53 is incapacitated by alterations of other pathway components [9]. The latter approach greatly expands the options in target selection for therapeutic intervention and the first studies performed with mouse models able to restore p53 activity by means of a genetic switch have shown that this strategy can lead to regression of tumours and increased survival of the animal [49-51]. Moreover, in some cases, the reactivation of the p53 pathway can be confined exclusively to cancer cells without affecting normal tissue, hence limiting side effects [7]. Among the different strategies for restoring p53 function, targeting the MDM2-p53 interaction by small molecules is one of the most investigated. Hence, one strategy to reactivate p53 in tumour types harbouring a wild-type p53 is to force the stabilization of p53 protein by liberating it from the negative control of MDM2. So far, several potential therapeutic agents able to interfere with the MDM2-p53 interaction by different mechanisms, such as Nutlins, benzodiazepines, RITA (Reactivation of p53 and Induction of Tumour cell Apoptosis), spiro-oxindoles and quinolinols, have been developed [27, 28, 30, 52-56]. In this frame it is noteworthy that the recovery of the cellular protein TRIM8 reactivates the p53 pathway upon drugs treatment in RCC cell line, because it prevents MDM2 binding to p53, as we have here shown.

In conclusion in the current study, we analyse for the first time how TRIM8 expression levels may influence the cellular response to chemotherapeutic drugs. We found that TRIM8 is dramatically down regulated in clear cell renal carcinoma (ccRCC), an aggressive drug-resistant cancer showing wild type p53. Interestingly, we showed that TRIM8 expression recovery in two different RCC cell lines renders these cells sensitive to chemotherapeutic treatments, strongly supporting the role of TRIM8 in strengthening the p53-mediated response.

Altogether our findings suggest that ccRCC can be successfully sensitized to conventional chemotherapy if combined with modalities designed to reactive p53, and more broadly TRIM8 could be considered a new target for therapeutic intervention in cancers where p53 is wild type and its pathway is defective.

## MATERIAL AND METHODS

### Cells and treatments

The human colon carcinoma cells HCT116, the human breast carcinoma cells MCF-7, the human osteosarcoma cells U2OS, the human proximal tubular epithelial cells HK-2 and the human renal cell carcinoma RCC Shaw and Elthem cell lines were cultured in Dulbecco's modified Eagle's medium (D-MEM) plus 10% foetal bovine serum (FBS), L-Glutamine (2 mM), penicillin (100 U/ml) and streptomycin (100 μg/ml) at 37°C, 5% CO_2_. Nutlin-3 10 μM (Cayman) and Cisplatin 7.5 μM (Sigma) was used for 24 hours. Elthem and RCC Shaw are primary RCC cell lines established from primary kidney tissue explants. RCC Shaw was kindly donated by Prof. Walter J. Storkus (Dept. of Immunology, University of Pittsburgh, PA, USA). Elthem cell line was patented by Prof. Elena Ranieri and currently provided by Public Health England (PHE) - culture collections (http://www.phe-culturecollections.org.uk).

### Transfections

5 × 10^5^ cells were plated 24h before transfection. At the time of transfections (60-80% cell confluence), 200 μl of D-MEM medium without serum were incubated with Trans-LT1 Mirus transfection reagent (Tema Ricerca) for 5 min at room temperature. Then, the empty pcDNA_3_-HA-vector (control), pcDNA_3_-HA-TRIM8, the empty pRS (control) or four different TRIM8 short hairpin RNAs (Origene™) were added to the medium containing the transfection reagent and incubated at room temperature for 20 min and subsequently added to the cell cultures for 48h.

### Cell proliferation assays by MTT reduction

1 × 10^5^ cells were plated in six-well plates. After treatments, 200 μl of MTT solution (5 mg/ml) were added to the cells for 4h at 37°C. The medium was then removed and the reduced blue formazan crystals were resuspended in isopropanol prior to reading the absorbance at 580 nm.

### Protein extraction from tissues and cell lines and Western blot analysis

Tissue samples were homogenate in ice-cold sample buffer (8M urea, 4% CHAPS, 40 mM Tris-base, 65 mM DTT containing a protease inhibitor cocktail) and after 30 minutes incubation on ice, samples were centrifuged at 13000 x g at 4°C. The surnatants were collected in new tubes and protein content was assayed by the Bradford dye-binding method (BioRad Protein assay).

Cells were plated in 100-mm culture dishes at a density of 5×10^5^ cells/ml. After treatments, cells were lysed and extracted as previously described [57].

For immunoblotting, the following primary antibodies were used: p53 specific DO-1 (Santa Cruz, California, USA 1:300), p53-Ser15P (Santa Cruz, California, USA 1:100), p53-Ser20P (Santa Cruz, California, USA 1:100), MDM2 specific 2A10 (Calbiochem 1:400), p21 specific C-19 (Santa Cruz, 1:200), TRIM8 specific C-20 (Santa Cruz, California, USA 1:200), Anti-HA (Bethyl Laboratories, 1:1000), Anti-Actin Ab-1 antibodies kit (Calbiochem, 1:2000). Bound primary antibodies were visualized using Lumi-Light Western Blotting Substrate (Roche™) on a UVITEC Cambridge Camera.

### Co-Immunoprecipitation and Western blot analysis

Co-immunoprecipitation experiments were performed by lysing RCC cells in RIPA buffer addicted with glycerol 10% to stabilize protein-protein interactions. Protein complexes were then immunoprecipited by using appropriate antibodies. Complexes were analysed by western blotting using appropriate antibodies, p53 specific DO-1 (Santa Cruz Biotechnology), anti-MDM2 (Calbiochem, Ab-2 2A10) and anti-HA (Bethyl Laboratories). Bound primary antibodies were antibodies were visualized using Lumi-Light Western Blotting Substrate (Roche™) on a UVITEC Cambridge Camera.

### DNA extraction from HK-2 and RCC cell lines

Genomic DNA from renal HK-2 and RCC cell lines was extracted using the QIAamp DNA Mini Kit (Qiagen^®^) according to the manufacturer's instructions. Extracted DNA was then quantified using the NanoDrop^TM^ 1000 Spectrophotometer (Thermo Scientific) and DNA quality was determined by running aliquots on the 2100 Bioanalyzer (Agilent Technologies).

### Gene dosage analysis

Two regions (region 1 - hg19 chr10:104409824-104409964; region 2 - hg19 chr10:104417047-104417193) of the TRIM8 gene and one region (hg19 chr3:189590756-189590945) of the TP63 gene of the HK-2 and RCC (both Shaw and Elthem) DNA were amplified by quantitative PCR using SYBR^®^ Select Master Mix (Life Technologies^TM^). qPCR reactions were carried out in triplicate using the ABI PRISM 7900HT platform (Applied Biosystems^®^, Life Technologies^TM^) using 50 ng of input DNA template for all cell lines. No template controls were included as negative controls for each primer pair (primers sequences are reported in Table 1). Amplification parameters were as follows: hot start at 95°C for 15 min; 50 amplification cycles (94°C for 15 sec, 62°C for 30 sec, 72°C for 30 sec); dissociation curve step (95° C for 15 sec, 60°C for 15 sec, 95°C for 15 sec). Fluorescence raw data were exported from the SDS 2.2.1 software (Applied Biosystems^®^, Life Technologies^TM^) and analysed with the DART-PCR Excel workbook [58]. Actual amplification efficiency values (E) for each amplicon were used to correct Cq values before analysing these data by the ΔCq method to compare relative gene quantity.

**TABLE 1:** Primer pairs for gene dosage analysis

Locus	Forward Primer (5′-3′)	Reverse Primer (5′ -3′)
chr10:104409824-104409964	TTGTCTGAAAACCTAGGG	GAGGCTTGGGGACTCTGG
chr10:104417047-104417193	CCGGCCACCAGGATTTCTAC	AAGGGCAGGTCTCTGGATGC
chr3:189590756-189590945	CAGCAGCACCAGCACTTACTTC	AAGGTTGCAACTGAAAGAGGG

Two-tailed Student's T tests were performed to assess the statistical significance of differences in DNA content for each locus analyzed. In this study, a p-value of less than 0.05 was considered to be statistically significant. Statistical analyses were performed by Analysis ToolPak in Microsoft Excel 2010 software.

### RNA extraction from patients and cell lines

Tumour and paired adjacent non-tumour renal parenchyma samples from a total of 24 patients were used for this work. Immediately after surgery, tissues were separately stored and frozen at -80°C according to a standard procedure. From histological examination, 20 samples were classified as clear cell RCCs (10 males and 10 females; mean age: 63 ± 10.8 years) and 4 were oncocytomas (all males; mean age: 63.25 ± 4.86 years) (Table 2).

**TABLE 2:** Anagraphic (gender and age) and clinical (Fuhrman grade) characteristics of patients affected by clear cell Renal Cell Carcinoma (ccRCC) and renal oncocytoma (RO)

Patient ID	Gender	Age	Grading
ccRCC_01	M	60	G2
ccRCC_02	F	69	G2
ccRCC_03	F	82	G2
ccRCC_04	F	72	G1
ccRCC_05	M	61	G2
ccRCC_06	M	78	G2
ccRCC_07	F	71	G3
ccRCC_08	M	59	G3
ccRCC_09	F	52	G3
ccRCC_10	M	46	G1
ccRCC_11	F	82	G1
ccRCC_12	M	54	G2
ccRCC_13	M	48	G1
ccRCC_14	M	68	G2
ccRCC_15	F	67	G3
ccRCC_16	F	59	G1
ccRCC_17	M	66	G2
ccRCC_18	F	55	G1
ccRCC_19	M	48	G2
ccRCC_20	F	63	G3
RO_01	M	68	
RO_02	M	66	
RO_03	M	57	
RO_04	M	62	

Furthermore, the pathological staging was determined according to the latest TNM classification and grading according to Fuhrman, as reported in Table 2. Informed consent to take part in this study was obtained from all the patients. The study was approved by the Hospital's Ethics Committee.

Collected ccRCC samples were processed for total RNA extraction from 50-100 mg of fresh frozen tissue using the TRIzol reagent (Invitrogen^TM^, Life Technologies^TM^). All RNA samples were purified using the RNeasy Mini kit (Qiagen^®^) according to the manufacturer's instructions. For HK-2 and RCC cell lines, total cellular RNA was extracted using the RNeasy mini kit (Qiagen). Purified RNA was then quantified using the NanoDrop^TM^ 1000 Spectrophotometer (Thermo Scientific) and RNA quality was determined by running aliquots on the 2100 Bioanalyzer (Agilent Technologies).

### qRT-PCR analysis

Reverse transcription of 500 ng of total RNA was performed using QuantiTect^®^ Reverse Transcription kit (Qiagen^®^). Control reverse transcription reactions without RT enzyme were also prepared and controlled by PCR for gDNA contamination.

Particular attention was paid to the choice of housekeeping genes. The geNorm VBA applet for Microsoft Excel was used to determine the most stable housekeeping genes for ccRCC and renal oncocytoma samples used in this study from a panel of five reference genes (ACTB, B2M, GAPDH, HPRT1 and RPL13) in 8 of the 18 ccRCC and all renal oncocytoma sample pairs used in qRT-PCR experiments, as previously described.^59^ For the geNorm analysis, qRT-PCR experiments were performed in duplicate on the ABI PRISM 7900HT platform (Applied Biosystems^®^, Life Technologies^TM^) using 1.5μl cDNA as template for each reaction with TaqMan^®^ Universal PCR Master Mix (Applied Biosystems^®^, Life Technologies^TM^). TaqMan assays from Applied Biosystems^®^ were used for the amplification of ACTB (Hs99999903_m1), B2M (Hs99999907_m1), GADPH (Hs99999905_m1), HPRT1 (Hs03929098_m1) and RPL13 (Hs00761672_s1) transcripts. No template controls (NTCs) were included as negative controls for each TaqMan assay.

Amplification parameters were as follows: hot start at 95°C for 10 min; 40 cycles of amplification (94°C for 15 sec, 60°C for 1 min). Results were first analysed in SDS 2.2.1 software and then exported in Microsoft Excel in order to be further analysed by using geNorm which outputted ACTB and RPL13 as best housekeeping genes for these samples.

qRT-PCR experiments were performed on ccRCC and renal oncocytoma samples and on HK-2 and RCC cell lines to measure TRIM8 expression by using TaqMan^®^ assay Hs00229451_m1. Expression levels were calculated relative to the mean expression levels of ACTB and RPL13 genes, according to the following formula: relative Expression Ratio (rER) = 2^{(Cq_TRIM8_ – [(Cq_ACTB_ + Cq_RPL13_)/2]}. The reported data represent the average of at least two independent experiments and are shown with their standard errors.

Two-tailed Student's T tests were performed to assess the statistical significance of gene expression levels differences observed between the normal and the ccRCC/RO samples (and also among the different grades of ccRCC) and the effects of Nutlin-3 or Cisplatin, in presence or in absence of TRIM8 over-expression, in HK-2 and RCC cells. In this study, a p-value of less than 0.05 was considered to be statistically significant.

## SUPPLEMENTARY MATERIAL FIGURES



## References

[1] Hanahan D, Weinberg RA (2000). The hallmarks of cancer. Cell.

[2] Riley T, Sontag E, Chen P, Levine A (2008). Transcriptional control of human p53-regulated genes. Nat Rev Mol Cell Biol.

[3] Hanahan D, Weinberg RA (2011). Hallmarks of cancer: the next generation. Cell.

[4] Toledo F, Wahl GM (2006). Regulating the p53 pathway: in vitro hypotheses, in vivo veritas. Nat Rev Cancer.

[5] Zambetti GP (2007). The p53 mutation “gradient effect” and its clinical implications. J Cell Physiol.

[6] Robles AI, Harris CC (2010). Clinical outcomes and correlates of TP53 mutations and cancer. Cold Spring Harb Perspect Biol.

[7] Lane DP, Brown CJ, Verma C, Cheok CF (2011). New insights into p53 based therapy. Discov Med.

[8] Shangary S, Wang S (2008). Targeting the MDM2-p53 interaction for cancer therapy. Clin Cancer Res.

[9] Cheok CF, Verma CS, Baselga J, Lane DP (2011). Translating p53 into the clinic. Nat Rev Clin Oncol.

[10] Canda AE, Kirkali Z (2006). Current management of renal cell carcinoma and targeted therapy. Urol J.

[11] Lam JS, Klatte T, Kim HL, Patard JJ, Breda A, Zisman A (2008). Prognostic factors and selection for clinical studies of patients with kidney cancer. Crit Rev Oncol Hematol.

[12] Tsao CC, Corn PG (2010). MDM-2 antagonists induce p53-dependent cell cycle arrest but not cell death in renal cancer cell lines. Cancer Biol Ther.

[13] Finley DS, Pantuck AJ, Belldegrun AS (2011). Tumor biology and prognostic factors in renal cell carcinoma. Oncologist.

[14] Takara K, Sakaeda T, Okumura K (2006). An update on overcoming MDR1-mediated multidrug resistance in cancer chemotherapy. Curr Pharm Des.

[15] Hodorová I, Rybárová S, Solár P, Vecanová J, Mihalik J, Bohus P (2008). Multidrug resistance proteins in renal cell carcinoma. Folia Biol (Praha).

[16] Warburton HE, Brady M, Vlatković N, Linehan WM, Parsons K, Boyd MT (2005). p53 regulation and function in renal cell carcinoma. Cancer Res.

[17] Zigeuner R, Ratschek M, Rehak P, Schips L, Langner C (2004). Value of p53 as a prognostic marker in histologic subtypes of renal cell carcinoma: a systematic analysis of primary and metastatic tumor tissue. Urology.

[18] Crispen PL, Boorjian SA, Lohse CM, Leibovich BC, Kwon ED (2008). Predicting disease progression after nephrectomy for localized renal cell carcinoma: the utility of prognostic models and molecular biomarkers. Cancer.

[19] Zubac DP, Bostad L, Kihl B, Seidal T, Wentzel-Larsen T, Haukaas SA (2009). The expression of thrombospondin-1 and p53 in clear cell renal cell carcinoma: its relationship to angiogenesis, cell proliferation and cancer specific survival. J Urol.

[20] Wittnebel S, Jalil A, Thiery J, DaRocha S, Viey E, Escudier B (2005). The sensitivity of renal cell carcinoma cells to interferon alpha correlates with p53-induction and involves Bax. Eur Cytokine Netw.

[21] Hutson TE, Thakkar S, Cohen P, Borden EC, Bukowski RM, Figlin RA, Motzer RJ (2009). Interferons and Interleukin-2: Molecular Basis of Activity and Therapeutic Results. Renal Cell Carcinoma.

[22] Caratozzolo MF, Micale L, Turturo MG, Cornacchia S, Fusco C, Marzano F (2012). TRIM8 modulates p53 activity to dictate cell cycle arrest. Cell Cycle.

[23] Hatakeyama S (2011). TRIM proteins and cancer. Nat Rev Cancer.

[24] Yuan Z, Villagra A, Peng L, Coppola D, Glozak M, Sotomayor EM (2010). The ATDC (TRIM29) protein binds p53 and antagonizes p53-mediated functions. Mol Cell Biol.

[25] Joo HM, Kim JY, Jeong JB, Seong KM, Nam SY, Yang KH (2011). Ret finger protein 2 enhances ionizing radiation-induced apoptosis via degradation of AKT and MDM2. Eur J Cell Biol.

[26] Sho T, Tsukiyama T, Sato T, Kondo T, Cheng J, Saku T (2011). TRIM29 negatively regulates p53 via inhibition of Tip60. Biochim Biophys Acta.

[27] Vassilev LT (2004a). Small-molecule antagonists of p53-MDM2 binding: research tools and potential therapeutics. Cell Cycle.

[28] Vassilev LT, Vu BT, Graves B, Carvajal D, Podlaski F, Filipovic Z (2004b). In vivo activation of the p53 pathway by small-molecule antagonists of MDM2. Science.

[29] Li X, Wang G, Zhao J, Ding H, Cunningham C, Chen F (2005). Antiproliferative effect of beta-elemene in chemoresistant ovarian carcinoma cells is mediated through arrest of the cell cycle at the G2-M phase. Cell Mol Life Sci.

[30] Vassilev LT (2007). MDM2 inhibitors for cancer therapy. Trends Mol Med.

[31] Qu K, Lin T, Wei J, Meng F, Wang Z, Huang Z (2013). Cisplatin induces cell cycle arrest and senescence via upregulating P53 and P21 expression in HepG2 cells. Nan Fang Yi Ke Da Xue Xue Bao.

[32] Forslund A, Zeng Z, Qin LX, Rosenberg S, Ndubuisi M, Pincas H (2008). MDM2 gene amplification is correlated to tumor progression but not to the presence of SNP309 or TP53 mutational status in primary colorectal cancers. Mol Cancer Res.

[33] Vogelstein B, Lane D, Levine AJ (2000). Surfing the p53 network. Nature.

[34] Sherr CJ, Bertwistle D, DEN Besten W, Kuo ML, Sugimoto M, Tago K (2005). p53-Dependent and -independent functions of the Arf tumor suppressor. Cold Spring Harb Symp Quant Biol.

[35] Haitel A, Wiener HG, Baethge U, Marberger M, Susani M (2000). Mdm2 expression as a prognostic indicator in clear cell renal cell carcinoma: comparison with p53 overexpression and clinicopathological parameters. Clin Cancer Res.

[36] Gurova KV, Hill JE, Razorenova OV, Chumakov P.M, Gudkov AV (2004). p53 pathway in renal cell carcinoma is repressed by a dominant mechanism. Cancer Res.

[37] Gurova KV, Hill JE, Guo C, Prokvolit A, Burdelya LG, Samoylova E (2005). Small molecules that reactivate p53 in renal cell carcinoma reveal a NF-kappaB-dependent mechanism of p53 suppression in tumors. Proc Natl Acad Sci U S A.

[38] Cambiaghi V, Giuliani V, Lombardi S, Marinelli C, Toffalorio F, Pelicci PG (2012). TRIM proteins in cancer. Adv Exp Med Biol.

[39] Petrera F, Meroni G (2012). TRIM proteins in development. Adv Exp Med Biol.

[40] Vincent SR, Kwasnicka DA, Fretier P (2000). A novel RING finger-B box-coiled-coil protein, GERP. Biochem Biophys Res Commun.

[41] Hudson CC, Liu M, Chiang GG, Otterness DM, Loomis DC, Kaper F (2002). Regulation of hypoxia-inducible factor 1alpha expression and function by the mammalian target of rapamycin. Mol Cell Biol.

[42] Linehan WM (2003). Molecular targeting of VHL gene pathway in clear cell kidney cancer. J Urol.

[43] Costa LJ, Drabkin HA (2007). Renal cell carcinoma: new developments in molecular biology and potential for targeted therapies. Oncologist.

[44] Linehan WM, Bratslavsky G, Pinto PA, Schmidt LS, Neckers L, Bottaro DP (2010). Molecular diagnosis and therapy of kidney cancer. Annu Rev Med.

[45] Ravi R, Mookerjee B, Bhujwalla ZM, Sutter CH, Artemov D, Zeng Q (2000). Regulation of tumor angiogenesis by p53-induced degradation of hypoxia-inducible factor 1alpha. Genes Dev.

[46] Roe JS, Youn HD (2006). The positive regulation of p53 by the tumor suppressor VHL. Cell Cycle.

[47] Roberts AM, Watson IR, Evans AJ, Foster DA, Irwin MS, Ohh M (2009). Suppression of hypoxia-inducible factor 2alpha restores p53 activity via Hdm2 and reverses chemoresistance of renal carcinoma cells. Cancer Res.

[48] Bartoletti-Stella A, Mariani E, Kurelac I, Maresca A, Caratozzolo MF, Iommarini L (2013). Gamma rays induce a p53-independent mitochondrial biogenesis that is counter-regulated by HIF1α. Cell Death Dis.

[49] Martins CP, Brown-Swigart L, Evan GI (2006). Modeling the therapeutic efficacy of p53 restoration in tumors. Cell.

[50] Ventura A, Kirsch DG, McLaughlin ME, Tuveson DA, Grimm J, Lintault L (2007). Restoration of p53 function leads to tumour regression in vivo. Nature.

[51] Jiang D, Brady CA, Johnson TM, Lee EY, Park EJ, Scott MP (2011). Full p53 transcriptional activation potential is dispensable for tumor suppression in diverse lineages. Proc Natl Acad Sci U S A.

[52] Issaeva N, Bozko P, Enge M, Protopopova M, Verhoef LG, Masucci M (2004). Small molecule RITA binds to p53, blocks p53-HDM-2 interaction and activates p53 function in tumors. Nat Med.

[53] Vazquez A, Bond EE, Levine AJ, Bond GL (2008). The genetics of the p53 pathway, apoptosis and cancer therapy. Nat Rev Drug Discov.

[54] Ahmed A, Yang J, Maya-Mendoza A, Jackson DA, Ashcroft M (2011). Pharmacological activation of a novel p53-dependent S-phase checkpoint involving CHK-1. Cell Death Dis.

[55] de Lange J, Verlaan-de Vries M, Teunisse AF, Jochemsen AG (2012). Chk2 mediates RITA-induced apoptosis. Cell Death Differ.

[56] Ma T, Yamada S, Ichwan SJ, Iseki S, Ohtani K, Otsu M (2012). Inability of p53-reactivating compounds Nutlin-3 and RITA to overcome p53 resistance in tumor cells deficient in p53Ser46 phosphorylation. Biochem Biophys Res Commun.

[57] Lefkimmiatis K, Caratozzolo MF, Merlo P, D'Erchia AM, Navarro B, Levrero M (2009). p73 and p63 sustain cellular growth by transcriptional activation of cell cycle progression genes. Cancer Res.

[58] Peirson SN, Butler JN, Foster RG (2003). Experimental validation of novel and conventional approaches to quantitative real-time PCR data analysis. Nucleic Acids Res.

[59] Vandesompele J, De Preter K, Pattyn F, Poppe B, Van Roy N, De Paepe A (2002). Accurate normalization of real-time quantitative RT-PCR data by geometric averaging of multiple internal control genes. Genome Biol.

