# Supplementary material for: TRIM8 anti-proliferative action against chemo-resistant renal cell carcinoma
Source: Oncotarget. 2014 Jun 8;5(17):7446–57. doi: 10.18632/oncotarget.2081 (PMC4202135; doi:10.18632/oncotarget.2081)
Supplement: Supplementary file 1 [file oncotarget-05-7446-s001.pdf]

TRIM8 anti-proliferative action against chemo-resistant renal cell carcinoma

Supplementary Material

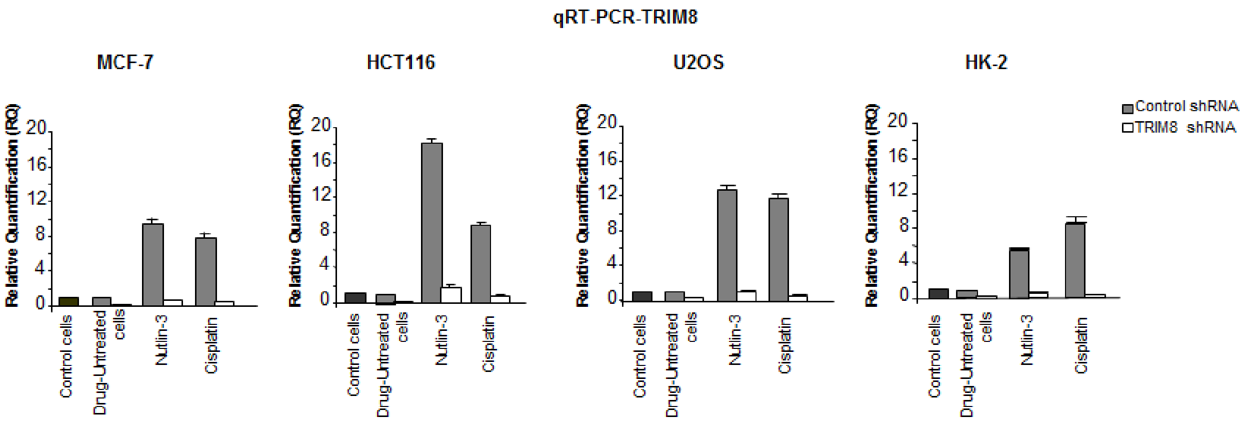

Supplementary Fig S1: Effects of TRIM8 depletion in different cell lines. qRT-PCR of TRIM8 in the HCT116, MCF-7, U2OS and HK-2 p53wt cell lines transfected for 48h with unspecific shRNA (control) or specific TRIM8-shRNAs and treated for 24h with the chemotherapeutic drugs Cisplatin (7.5 µM) or Nutlin-3 (10 µM).

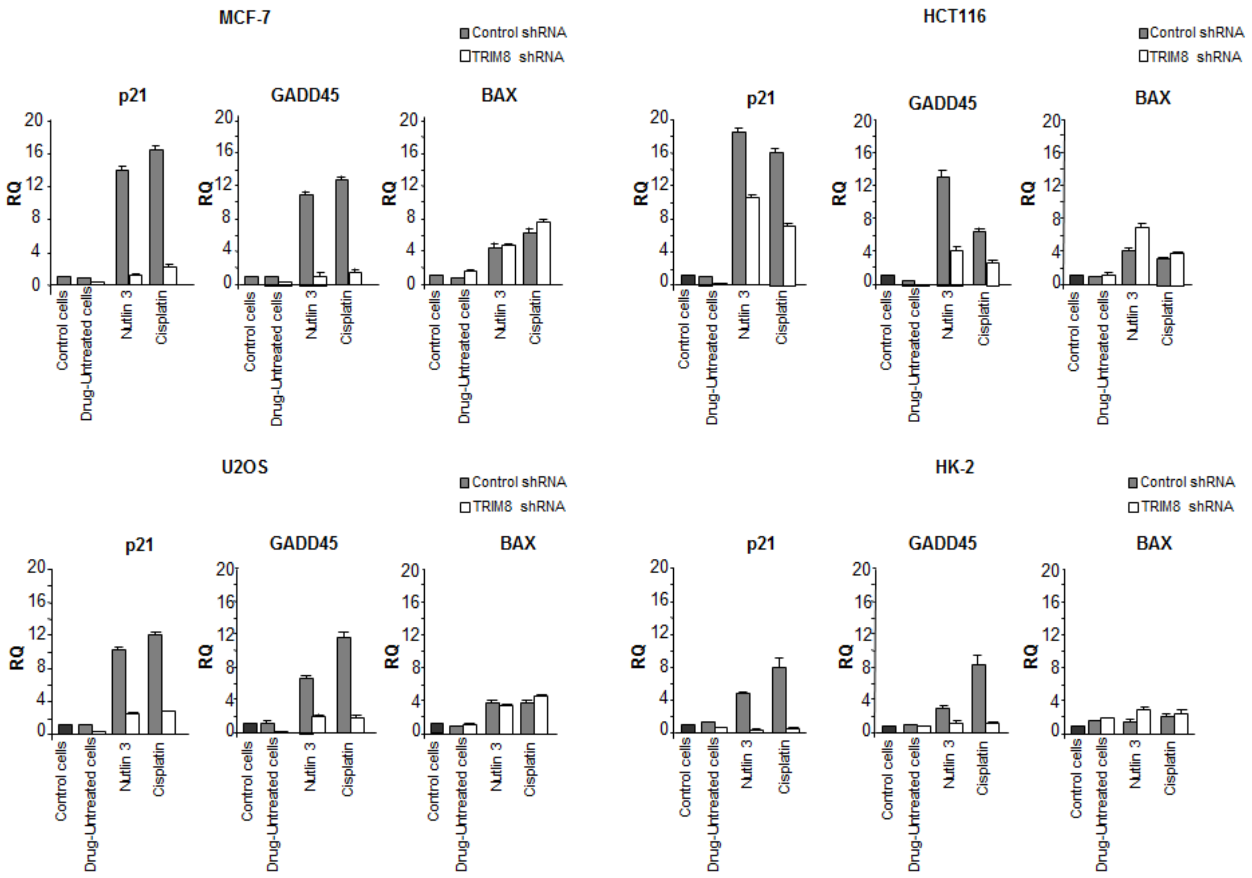

Supplementary Fig S2: Effects of TRIM8 depletion combined with chemotherapeutic drugs on p53 target genes expression. qRT-PCR of the indicated p53 target genes in MCF-7 and HCT116 p53wt and in H1299 and HCT116(p53-/-) p53 null cells transfected for 48 h with unspecific shRNA (control) or specific TRIM8-shRNAs and treated for 24h with the chemotherapeutic drugs Cisplatin (7.5 μM) or Nutlin-3 (10 μM).

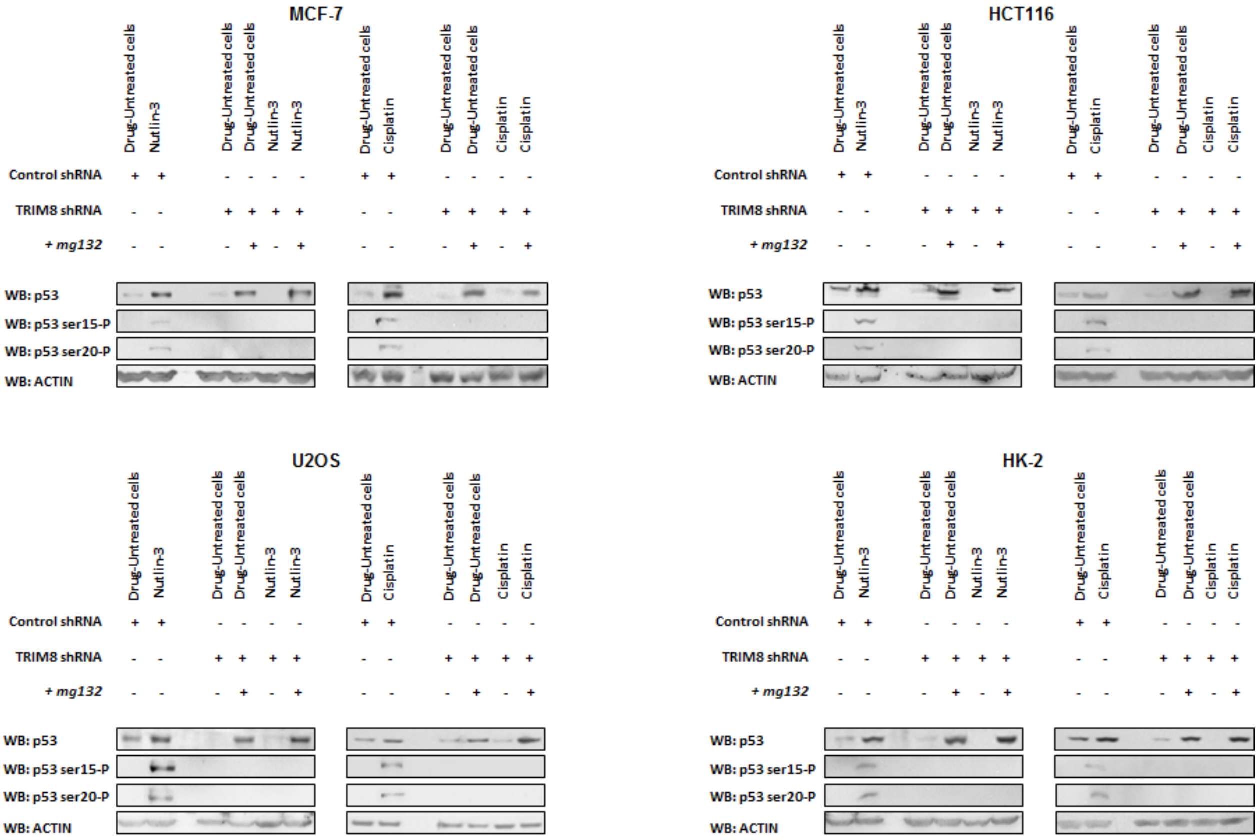

Supplementary Fig S3: Effects of TRIM8 depletion combined with chemotherapeutic drugs on p53 phosphorylation. Western blotting analysis of the indicated proteins 48h after transfection with unspecific shRNA (control) or specific TRIM8-shRNAs and 24h after treatment with the chemotherapeutic drugs (Cisplatin 7.5 µM or Nutlin-3 10 µM). Western blot of Actin was conducted as control.

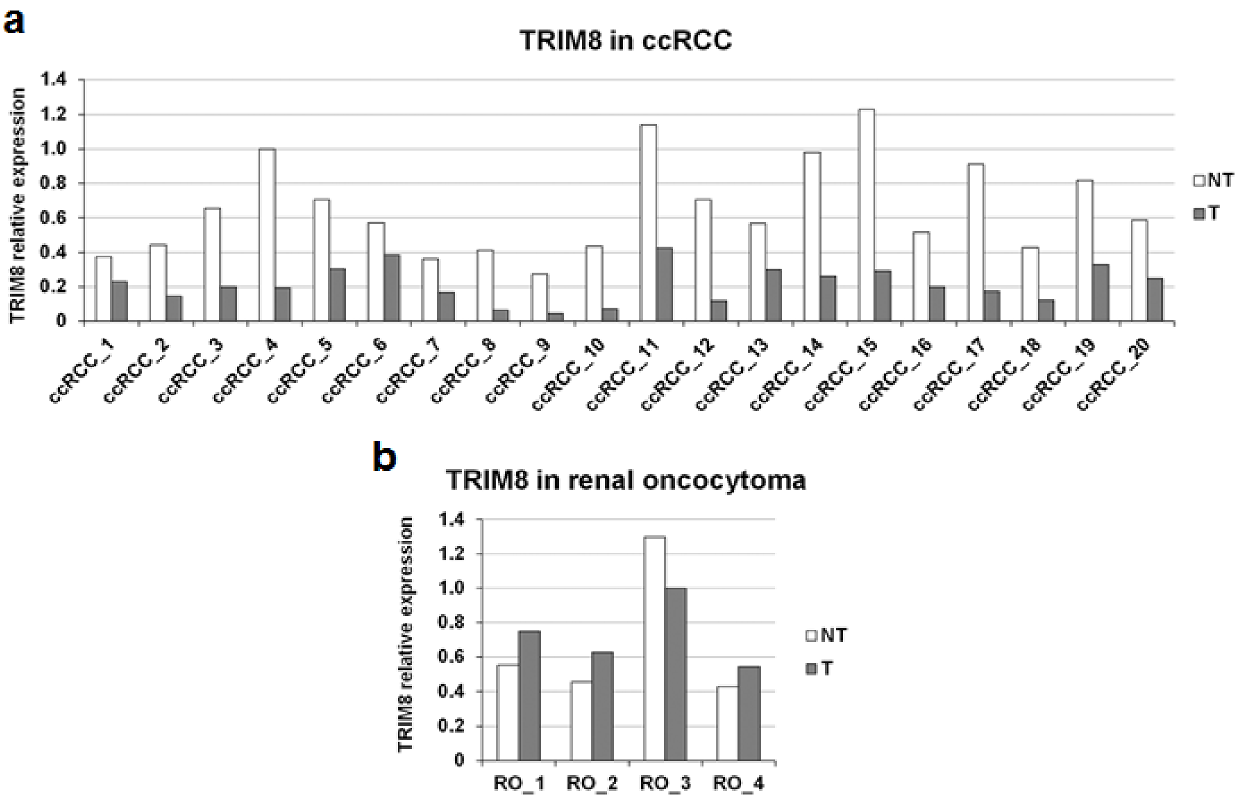

Supplementary Fig S4: TRIM8 expression in renal cancer samples. TRIM8 mRNA expression for the 20 ccRCC samples (T) **(a)** and each of the 4 renal oncocytoma (T) **(b)** and and their paired non-tumor tissues (NT). Expression data were measured respect to one normal sample chosen arbitrarily as calibrator and then normalized by the geometric mean of ACTB e RPL13 expression ratios. * p-value < 0.001.
